# The monkeypox outbreak in 2022: adaptive evolution associated with APOBEC3 may account for

**DOI:** 10.1038/s41392-022-01181-x

**Published:** 2022-09-16

**Authors:** Yangzhen Chen, Maochen Li, Huahao Fan

**Affiliations:** College of Life Science and Technology, Beijing University of Chemical Technology, Beijing, China

**Keywords:** Microbiology, Infectious diseases, Infection

In a recent study published on *Nature Medicine*, Isidro, J et al. attempted to explore the potential causes of the recent monkeypox outbreak in 2022,^1^ which has been declared as a Public Health Emergency of International Concern (PHEIC). The genetic characterization study based on shotgun metagenomics suggested that the mutational bias (from GA to AA or TC to TT) in monkeypox genomes driven by apolipoprotein B mRNA-editing catalytic polypeptide-like 3 (APOBEC3) enzyme might account for the PHEIC.

On July 1 and July 24, 2022, two groups reported the epidemiologic investigation and clinical characteristics of recent monkeypox (MPX) epidemics in *Lancet Infectious Diseases* and *The New England Journal of Medicine* (NEJM), respectively.^2,3^ In the report published in *Lancet Infectious Diseases*, among the 54 MPX cases, 44 (82%) patients had different degrees of prodromal symptoms and 13 (24%) were HIV-infected.^2^ In the multinational research involving 43 sites in 16 countries between April 27 and June 24, 2022 published in NEJM, most of the 528 monkeypox patients presented systemic clinical features with fever (62%), lethargy (41%), myalgia (31%), and headache (27%), and 41% of them are human immunodeficiency virus (HIV) infected.^3^ It is notable that the majority of monkeypox cases reported in both two studies are homosexual or bisexual men (54 of 54 patients and 519 of 528 patients, respectively), with a high proportion of rash, anogenital lesions, and sexually transmitted infection (STI),^2,3^ which provoked discussions about whether monkeypox is a kind of sexually transmitted disease besides the route of face-to-face contact.^4^

**Fig. 1:**
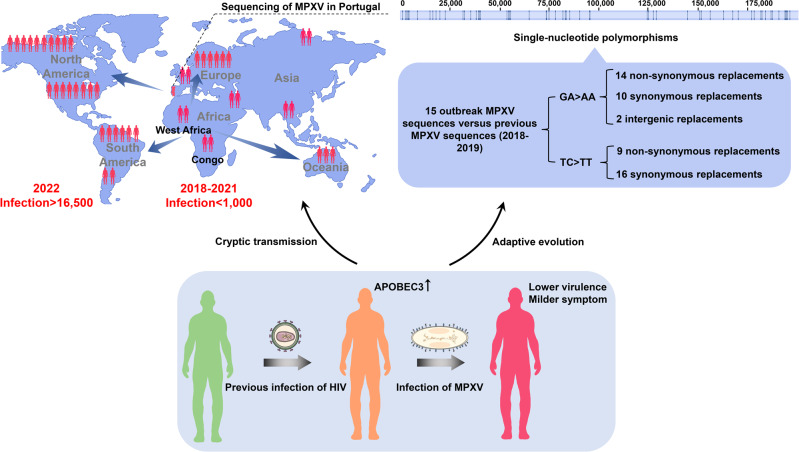
APOBEC3-associated virus adaptive evolution may account for the monkeypox outbreak in 2022. The historical epidemiology and the genetic linkages suggested that the global monkeypox outbreak in 2022 might originate from epidemic regions (Africa). Sequencing analysis identified nearly 50 biased mutations (from GA to AA or TC to TT) in the genomes of recent monkeypox epidemics, indicating an accelerated evolution driven by APOBEC3. Coincidently, 218 (41%) of 528 monkeypox cases in the study of J.P. Thornhill et al. are also HIV patients with a high level of APOBEC3 expressions, which is beneficial for MPXV-biased mutations. The APOBEC3-derived mutations may further reduce the pathogenicity and symptoms caused by MPXV infection, leading to the cryptic transmission of monkeypox in populations and the global monkeypox outbreak, exhibiting the adaptive evolution of MPXV

Monkeypox virus (MPXV) infection can cause some influenza-like symptoms, with fever, fatigue, lethargy, or myalgia, progression to pathognomonic skin lesions containing pustular papules, fluid-filled vesicles, and ulcerations, and may present with lymphadenopathy. There are 94 and 73% of monkeypox patients, respectively, in the above studies presented different degrees of skin lesions on the genital or perianal skin rather than lesions over the entire body reported in previous cases.^2,3^ The lesions in these uncommon parts of the body resulted in treatment delays in 377 patients being misdiagnosed as venereal diseases,^2^ which may provide clues to explain that most patients are men who have sex with men (MSM) in this outbreak. The observational analysis from British sexual health clinics in London also found that among 52 monkeypox cases, there are 49 patients had sex without condoms, and 47 patients had more than five sexual partners in the short term, suggesting monkeypox might transmit through skin-to-skin or mucosal contact.^3^ Besides, milder symptoms with fewer skin lesions were observed in the monkeypox patients of this outbreak compared with previous cases reported in Africa,^2^ partly leading to less attention paid on some early cases, thus the monkeypox transmission in populations may have cryptically lasted for a long time. These explain why more than 16,500 cases have been reported in at least 74 countries during the last 2 months, compared with less than 1000 cases reported last 4 years (mainly occurred in Africa) (Fig. 1) (www.who.int). However, the reasons for the sudden monkeypox outbreak and the milder symptoms observed in the recent epidemic remain to be determined.

On June 24, 2022, a study published in *Nature Medicine* attempted to explain why monkeypox suddenly broke out worldwide in the last 2 months. Based on phylogenetic analysis, the first 15 published sequences of the 2022 MPXV outbreak were associated with the West African (WA) clade, which commonly causes epidemic diseases in the remote regions of West Africa with milder symptoms and lower death rate, compared with the Congo Basin (CB) clade.^1^ The historical epidemiology and the genetic linkages suggested that the sudden global monkeypox outbreak in 2022 might originate from epidemic regions, since the MPXV sequences in 2022 all have genetic relevance to those of the monkeypox outbreak in Nigeria and the spillover through international travel in 2018–2019.^1^

Notably, about 50 genetic differences were identified in the viral genomes of recent cases compared with those detected in 2018–2019, especially within three amino acid changes (D209N, P722S, and M1741I) in the surface glycoprotein B21.^1^ B21 is an important immune target and its mutation is beneficial for virus immune evasion and transmission. Moreover, there were 46 single-nucleotide polymorphisms (SNPs) presenting mutational bias, with 26 and 15 replacements with GA > AA and TC > TT, respectively.^1^ The unusual mutational bias and the characterization of abundant A: T bases in MPXV DNA implied the possibility of a non-random driver such as Apolipoprotein B mRNA-editing catalytic polypeptide-like 3 (APOBEC3). As one of the DNA cytidine deaminase, APOBEC3 is the only one whose expression is remarkably upregulated after HIV infection^5^, inhibiting virus replication by mediating the viral genome mutations, which is a part of innate immunity in mammals. APOBEC3 has been identified the antiviral activity against viruses such as HIV, hepatitis B virus (HBV), human papilloma virus (HPV), herpes simplex virus (HSV), and epstein-barr virus (EBV), and can also be upregulated during infection.^6^ Meanwhile, it was found that 218 (41%) of 528 monkeypox cases in the study of J.P. Thornhill et al., are also HIV patients with a high level of APOBEC3 expressions, which is beneficial for MPXV-biased mutations.^1,7^ The specific mutations driven by APOBEC3 may further reduce the pathogenicity and symptoms caused by MPXV infection, facilitating the cryptic transmission of monkeypox in populations, and suggesting an adaptive evolution.

Although the adaptive mutations have been found in the genomes of recent monkeypox outbreaks, MPXV, as a DNA virus, theoretically will not frequently mutate like RNA viruses such as SARS-CoV-2, and the continuous MPXV sequence surveillance is still deserved in the context of the worldwide monkeypox outbreak. Previous smallpox vaccination is believed to provide cross-protection against MPXV infection to some degree, thus healthcare workers and high-risk groups are encouraged to be vaccinated to prevent onward infections, known as a “ring vaccination” strategy. Furthermore, two smallpox drugs, cidofovir and tecovirimat, approved by the United States Food and Drug Administration (FDA) were proved effective for monkeypox treatment in clinical trials, and it is not necessary for the public to panic.
